# High speed e-beam lithography for gold nanoarray fabrication and use in nanotechnology

**DOI:** 10.3762/bjnano.5.202

**Published:** 2014-10-30

**Authors:** Jorge Trasobares, François Vaurette, Marc François, Hans Romijn, Jean-Louis Codron, Dominique Vuillaume, Didier Théron, Nicolas Clément

**Affiliations:** 1 Institut d’Electronique Microélectronique et Nanotechnologie (IEMN) CNRS, Avenue Poincaré, 59652, Villeneuve d’Ascq, France; 2 Vistec Lithography BV, De Dintel 27a, 5684 PS Best, The Netherlands

**Keywords:** gold nanodot, gold nanoparticle, high-speed e-beam lithography, molecular electronics, nanoarray, self-assembled monolayers, XPS

## Abstract

E-beam lithography has been used for reliable and versatile fabrication of sub-15 nm single-crystal gold nanoarrays and led to convincing applications in nanotechnology. However, so far this technique was either too slow for centimeter to wafer-scale writing or fast enough with the so-called dot on the fly (DOTF) technique but not optimized for sub-15 nm dots dimension. This prevents use of this technology for some applications and characterization techniques. Here, we show that the DOTF technique can be used without degradation in dots dimension. In addition, we propose two other techniques. The first one is an advanced conventional technique that goes five times faster than the conventional one. The second one relies on sequences defined before writing which enable versatility in e-beam patterns compared to the DOTF technique with same writing speed. By comparing the four different techniques, we evidence the limiting parameters for the writing speed. Wafer-scale fabrication of such arrays with 50 nm pitch allowed XPS analysis of a ferrocenylalkyl thiol self-assembled monolayer coated gold nanoarray.

## Introduction

Well-ordered arrays of nanoparticles are already showing exciting applications in nanotechnology, including materials science [1–5], electronics [6–10], biology [11–14] and information technology [14–15]. Combined top-down/bottom-up fabrication with versatile and well-controlled fabrication of gold nanoarrays coupled with (bio)molecules self-assembly offer great promises for fundamental research on molecular electronics [4,8] or high-throughput screening based on single-biomolecule arrays [12]. However, the top-down approach using e-beam lithography is actually too slow for fabricating dense gold nanoarrays at cm^2^-scale, which precludes use of these technologies for some applications (mainly optics) or for chemical characterization (such as XPS). Typically, fabrication of 1 cm^2^ nanoarray of 10 nm gold NPs with 100 nm pitch requires 4 days of e-beam writing [1]. To overcome this problem, several alternative techniques are proposed [2,16–18]. The diblock-copolymer approach that consists of two chemically different polymer chains (or blocks) joined by a covalent bond is one of the most promising methods for low-cost and high speed fabrication of such gold nanoarrays [18]. However, to keep the versatility, well positioning and reliable nanoarray fabrication offered by e-beam lithography, another way is to notice that high-speed e-beam writing can be specifically developed for such nanoarray fabrication. Such high-speed e-beam technique called “dot-on-the-fly” (DOTF) has been previously developed for 25 nm diameter periodic metal patterns fabrication [19] and more recently for making 14 nm diameter holes for thermoelectricity application [20]. DOTF technique is however restricted to rectangle patterns. Here, we demonstrate that gold nanoarrays of sub-15 nm diameter, 50 nm pitch can be successfully fabricated either by the DOTF technique or by a new technique called “sequence method” allowing us XPS characterization of ferrocene-thiolated gold NPs prior molecular electronics study. We also propose an “advanced” conventional technique and discuss quantitatively the limiting parameter for each technique.

### Conventional and fast e-beam fabrication of gold nanoarrays

The usual strategy for making these gold nanoarrays using e-beam lithography is to open nano-holes in a positive resist (see experimental section for details), evaporate gold and remove the resist with lift-off. The gold evaporation step is of great importance because the gold implantation inside the silicon substrate together with the diffusion process allows the formation of perfect gold nanocrystals after annealing (ideal truncated octahedron or cuboctahedron nanoparticles (NPs)) [1,4]. In addition, these NPs have an ohmic contact for the bottom electrode, which is of great importance for molecular electronics applications. For example, in [8], a conducting AFM tip (CAFM) is used as a top electrode and the gold nanocrystals act as bottom electrodes. Within a single CAFM image it is possible to get statistics on thousands of molecular junctions which allowed us, in particular, to evidence the presence of 2 phases of organization on alkyl-thiolated gold nanoparticles. In order to study more functional molecular junction (for example redox molecules), we need few mm-large gold nanoarray for chemical characterization with usual techniques such XPS. This requires high-speed e-beam lithography.

**Figure 1:**
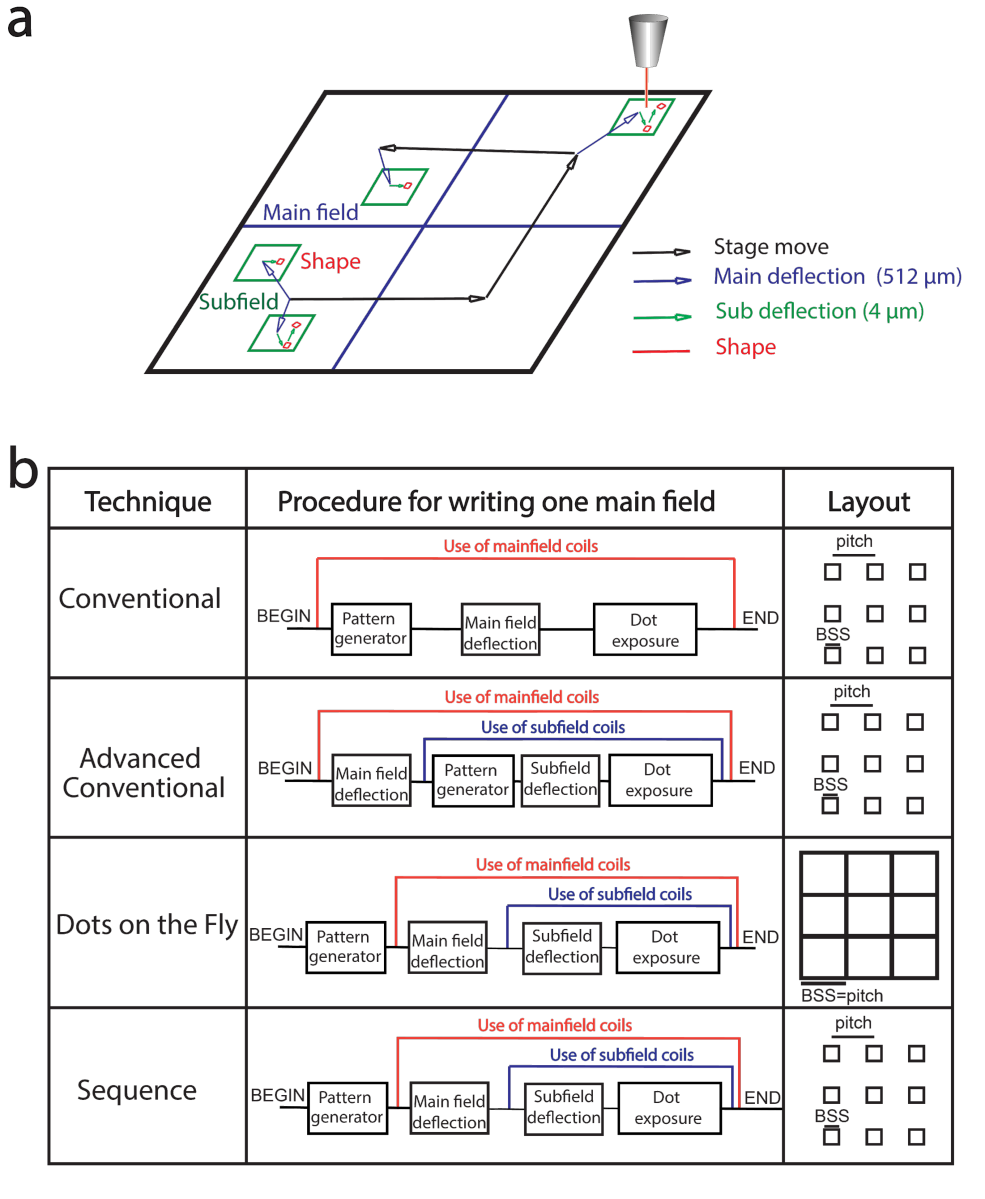
a) Schematic description of the writing strategy in e-beam lithography. The beam is deflected into a main field (≈512 µm) thanks to 2 sets of scanning coils, and to write a complete pattern, the stage moves from one main field to the other one. b) Schematic description of the 4 e-beam lithography techniques compared for their writing sequence inside a main field and their layout (BSS is the beam step size).

We first describe the relevant e-beam operation mechanisms (Figure 1a) that include specific parameters related to our e-beam writer (Vistec EBPG 5000+ operating at 100 keV, 20 bits). As the beam cannot be deflected over several mm, the layout has to be divided into main fields of up to 512 µm square at maximum. Inside this main field, the beam is deflected thanks to two sets of scanning coils: the main field coils and the subfields coils. Eventually, the efficient use of subfields (4 µm square at maximum) can lead to faster e-beam writing since settling times of subfield and mainfield coils are typically 0.5 µs and 40 µs, respectively. The procedure (path and exposure time) used by the e-beam is generated in real time using a pattern generator. This pattern generator is called for each shape (here we call shape one design to be written). This step, that also consumes time, can be optimized. The stage is moving from one main field to the other one for the complete layout writing. Below, we describe each of the proposed method for nanoarray fabrication (Figure 1b) and discuss their performance in the next section.

In the conventional method, we design a grating of 5 nm by 5 nm nanodots equivalent to the beam step size (BSS) of the machine. Thus, each dot is equivalent to a shape consisting on one pixel and then moved to the next shape again consisting of one pixel. Since each dot is considered as an independent shape, the pattern generator is called for each dot. The e-beam exposes the first dot with the desired dose and then moves to the second dot using the main field deflection. The process is repeated for the other dots. Another method presented, that we call “advanced conventional method”, uses the subfield coils instead of the mainfield coils inside one subfield. In this last method, main coils are only used to go from one subfield to another one. Both of the conventional methods call the pattern generator for each dot because one dot is considered as one shape.

To overpass the pattern generator limitation, alternative high speed writing techniques are emerging. Firstly, with the called “dots on the fly” (DOTF) approach [19–20], compatible with the use of sub-fields, the array of dots layout is simplified to a single “big square” so as to generate the pattern only once. The main idea is that each pixel corresponds to the distance between dots. Technically, this can be achieved by increasing the BSS to the exact distance between dots (Figure 1b). This technique works because the beam dimension is around 10 nm whatever the BSS. This “big square” pattern, however, limits the patterns to rectangular array of dots within a single exposure. For example, the triangular structure, of importance to optimize the density of dots, may only be obtained by aligning several layouts.

The last method called “sequence method” that we introduce here for the gold nanodots array fabrication defines shapes as a series of lines and jumps with either beam “on” or “off” (See Supporting Information File 1 for the detailed code). In this way it is possible to define many dots as a single shape (ideally its dimension is that of a subfield) which can be repeated. As a consequence this method also reduces drastically the call to the pattern generator but with the additional flexibility to define the geometry of the shape (for example triangular array). Below, we demonstrate its efficiency for nanoarray fabrication.

## Results and Discussion

### Fabricated dots

**Figure 2:**
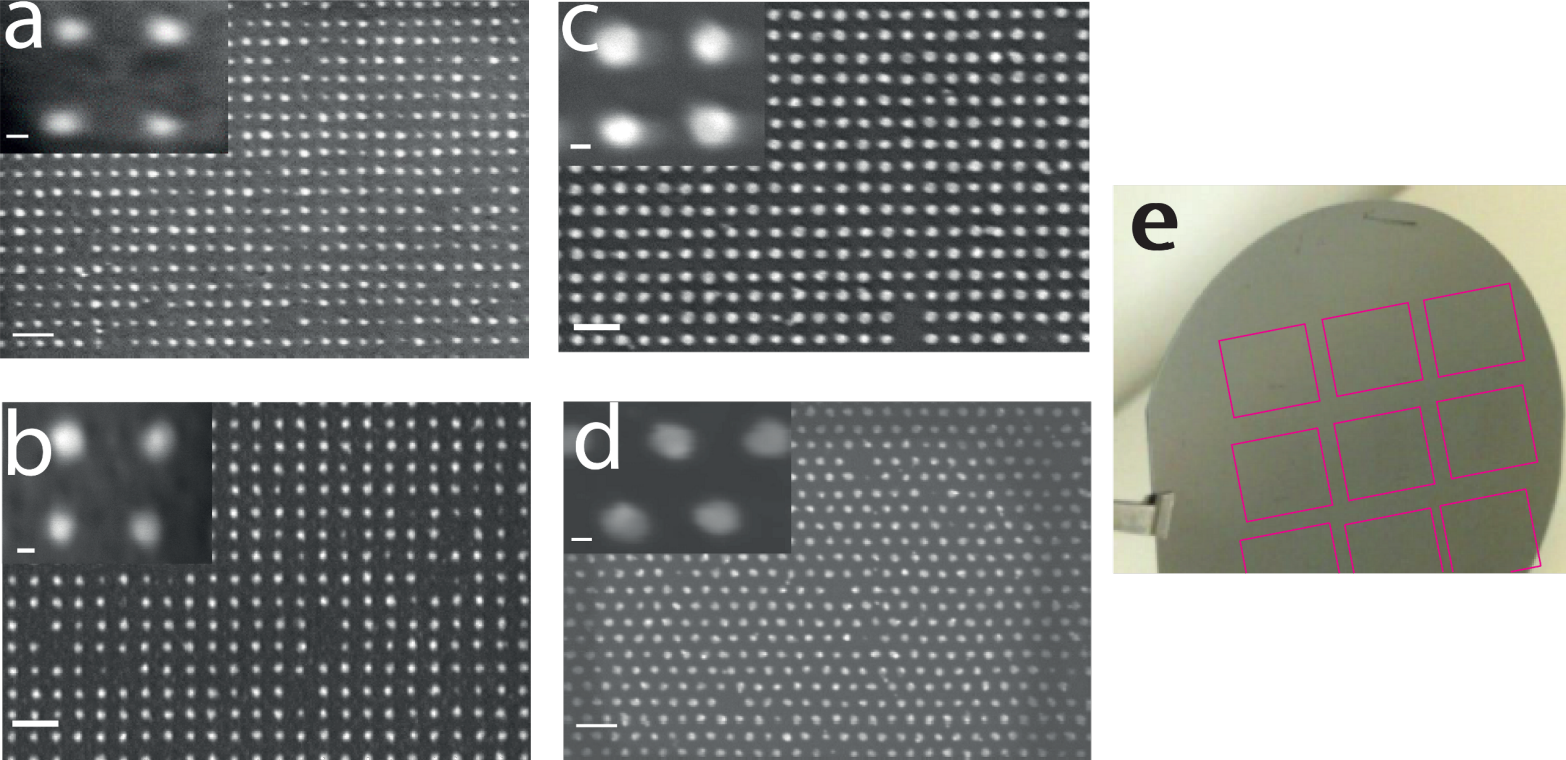
Scanning electron microscope (SEM) images of the gold nanodot arrays fabricated by (a) the “conventional” method with an exposure dose of 16000 µC/cm^2^ (4 fC/dot), (b) the “advanced conventional” method with an exposure dose of 16000 µC/cm^2^ (4 fC/dot) (c) the DOTF method with an exposure dose of 160 µC/cm^2^ (4 fC/dot) (d) the “sequence” method with an exposure dose of 12000 µC/cm^2^ (3.5 fC/dot). The beam current is set to 10 nA for the three techniques. The scale bar is 100 nm for the 4 SEM images and 10 nm for zoomed SEM images shown in inset. (e) Picture of a 3 inch wafer where 9 sequences of 1 cm^2^ have been written using DOTF and “sequence” methods. Given the small contrast provided by the 8 nm thick gold nanoparticles, these arrays are indicated by pink squares.

Figure 2 shows scanning electron microscope (SEM) images of the gold nanoarray (dots 10–15 nm, pitch = 50 nm) fabricated by the “conventional” (Figure 2a), the “advanced conventional” (Figure 2b), the DOTF (Figure 2c) and the “sequence” (Figure 2d) techniques. We didn’t notice significant difference in the fabricated gold nanoarrays. The dose per dot, corresponding to optimized nanodots (meaning less than 5% of missing dots and dots size below 15 nm), is similar for all the studied techniques: 3–4 fC/dot.

### Comparison of e-beam writing time for the four methods

**Figure 3:**
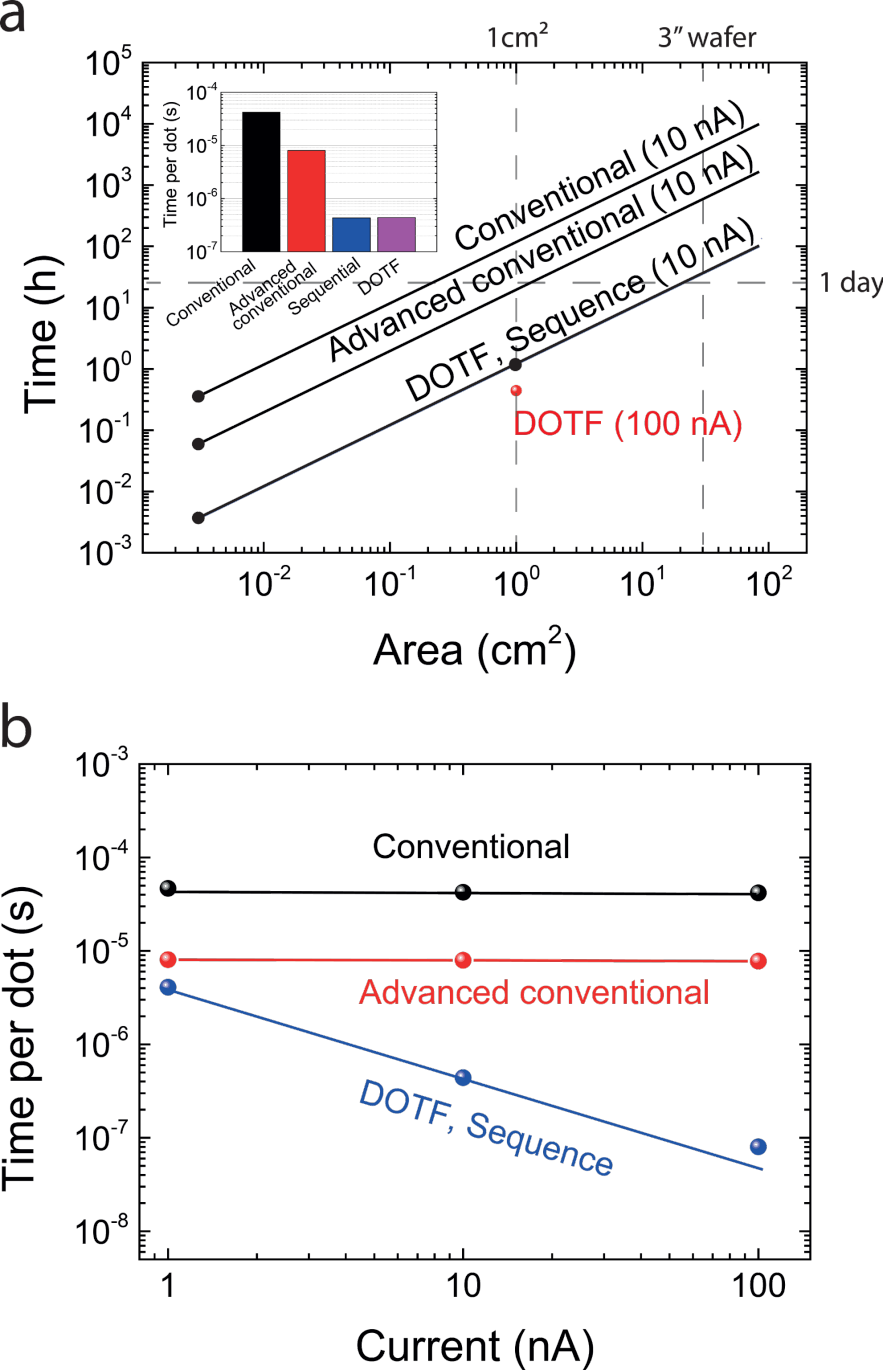
a) Plot of the estimated writing times per gold nanoarray area for each of the four different methods using an e-beam current of 10 nA and a pitch of 100 nm. Black points correspond to measured values. The writing time for DOTF technique with 100 nA is plotted for discussion. Inset: Equivalent writing time per dot for the four techniques. (b) Time per dot plotted as a function of the e-beam current for the four methods: ≈40 µs for the conventional method (limiting factor is the main field deflection) and ≈8 µs for the advanced conventional method (limiting factor is the pattern generator overhead). There is almost no overhead with DOTF and “sequence” methods, so the time is linear with the exposure time (inversely proportional to the current).

The inset of the Figure 3a shows the writing time normalized per dot for the four different techniques at a given e-beam current of 10 nA. We see a gain of two orders of magnitude in writing speed with both DOTF and “sequence” methods when compared to the conventional approach. Experimentally measured writing times for patterned square of width of 500 µm and 1 cm for high-speed lithography (100 nm-pitch) give a perfect match with the time per area (black points in Figure 3a). We can thus extrapolate time as a function of the size for all the techniques. Therefore, we plot in Figure 3a the estimated writing time as a function of nanoarray area for the four techniques. Whereas it would take 7 months for full wafer writing with the conventional method, it can take only ≈2 days with high-speed e-beam lithography. For molecular electronics application, chemical characterization of self-assembled monolayer coated gold nanoparticles is of prime importance, but it could not be achieved in [4,8] because a 1 cm^2^ nanoarray is required for comfortable XPS analysis. Whereas it would have required almost one full week of writing, we have written such nanoarray in less than 2 hours. This time can even be reduced to 25 minutes (and to 17 hours for a full 3” wafer) if an e-beam current of 100 nA is selected with the high speed techniques (Figure 3a). A systematic study of the influence of e-beam current on the writing time per dot *t*_exp/dot_ is shown in Figure 3b for the conventional and high speed techniques. These results can be satisfactorily explained with Equation 1:

[1]
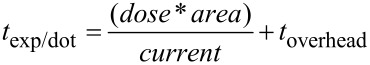


The first term corresponds to the exposure time and the second term to overhead time that includes mainfield, subfield settling times, pattern generator overhead and beam blanker. In the conventional method, the overhead time, mainly due to the mainfield settling (≈40 µs), is the limiting parameter (time per dot is equal to 46.6 µs, 42.3 µs and 41.7 µs for 1 nA, 10 nA and 100 nA, respectively). For the “advanced conventional method”, that uses the subfield coils, the time per dot has been reduced down to ≈8 µs. As the settling time of the subfield coils are typically in the order of 500 ns, we attribute this overhead time to the pattern generator. On the opposite, with the DOTF technique, *t*_overhead_ is negligible and a reasonable agreement with the experimental curve is obtained considering for example an e-beam current of 10 nA, an exposed area of 100 nm × 100 nm (corresponding to the distance between dots as explained previously) and a dose of 40 µC/cm^2^ (also equivalent to 4 fC per dot). Interestingly simple linear dependence, proposed in Equation 1, matches relatively nicely because the limitation parameter is the exposure time. With this technique, the pattern generator overhead is not present anymore because only one shape is sent to the pattern generator at the beginning of the writing (see Figure 1b). As we increase the e-beam current to large values (≈100 nA), higher dose should, however, be considered due to an increased spot diameter. We also noticed an increase in dot size to ≈30 nm diameter. For the “sequence” method, the approach is basically the same as with the DOTF technique: define many dots as a single shape which can be repeated. This enables to reduce drastically the overhead for settling times of the beam by reducing the number of shapes. It has the additional flexibility to define the geometry of the shape (e.g., triangular array). The corresponding writing time is therefore exactly the same as for the DOTF technique.

As a consequence the actual limitation of the proposed high-speed writing technique is the resist exposure time. Recently, direct patterning of high density sub-15 nm gold dot array using ultrahigh contrast electron beam lithography process on positive tone resist has been demonstrated [21]. Combination of high contrast resist and high speed writing e-beam lithography may further improve nanoarray fabrication’s speed.

### XPS measurements

**Figure 4:**
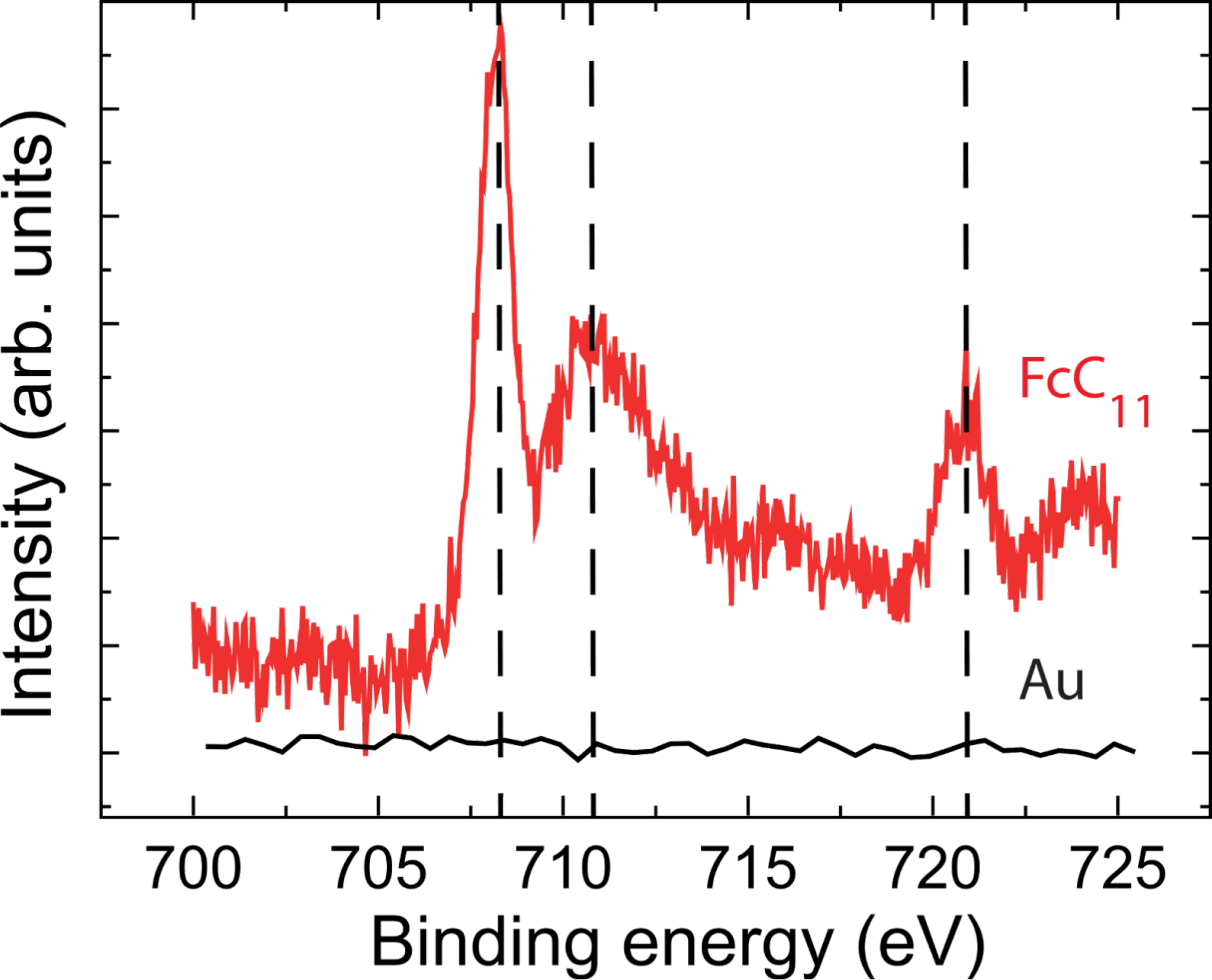
Fe 2p XPS spectra corresponding to the bare Au nanoarray and 11-ferrocenyl-1-undecanethiol (FcC_11_) SAM from bottom to top.

Using high-speed e-beam lithography with the “sequence” method, we have fabricated triangular nanoarrays of 1 cm^2^ with 50 nm pitch (Figure 2d) to optimize the area with useful signal (dots) for XPS characterization. Details for XPS measurements can be found in the Experimental section. We have selected the Ferrocene-thiol electroactive molecule, an important model system for the formation of electronic devices based self-assembly and biological sensors. XPS spectra have been well studied for such SAMs on a gold substrate, which allows a direct comparison with the literature. A self-assembled monolayer (SAM) of 11-ferrocenyl-1-undecanethiol (FcC_11_)-coated gold nanoarray was characterized by XPS (Figure 4).

The bare Au nanoarray shows no Fe 2p XPS signal confirming that the initial conditions prior to SAM formation corresponds to clean Au surface. XPS spectrum corresponding to the SAM with ferrocene (FcC_11_) show a Fe 2p_3/2_ and Fe 2p_1/2_ doublet located at 707.8 eV and 720.7 eV. The position of the doublet is in excellent agreement with previously reported values for adsorbed ferrocene [22–23]. Even more interestingly, we see a doublet of the Fe 2p_3/2_ peak that corresponds to the signal of Fc at 707.8 eV and Fc in its reduced state given that the signal of the ferricinium cation is expected at 710.6 eV [23–24]. As a consequence, ferrocene and ferricinium species coexist on our gold nanoarray [24]. Among the possible origins of the oxidation of the ferrocene into ferricinium [24], the exposure to light, although minimized, is the most likely in our sample.

## Conclusion

Gold nanoarrays with an ohmic contact to an highly doped silicon substrate fabricated by e-beam lithography have been proposed as a novel technique for molecular electronics study [1,4,8]. However, chemical characterization of the grafted molecules on these gold NPs could not be achieved because of the small patterned area limited by the e-beam writing time. Here, we have shown that a gain of 2 orders of magnitude in writing speed could be achieved for 10 nm dots and 3 orders of magnitude for 30 nm dots by optimizing a technique called “dot-on-the fly” and proposing a new “sequence” technique. A simple equation, proposed to explain the various parameters coming into play for e-beam writing, gave good agreement with our experimental datas. Using the “sequence” technique, we could successfully fabricate triangular pattern nanoarrays with dots every 50 nm at cm^2^-scale and obtain the XPS spectrum of a ferrocenylalkylthiol-coated nanoarray. We found that ferrocene and ferricinium (oxidized state) coexist after the self-assembly process. The developed technology, which will surely be of great importance for molecular electronics study on such nanoarrays, also promising exciting future works in chemistry and biosensing.

## Experimental

### Nanoarray fabrication

As described in [1,8], for e-beam lithography, we use an EBPG 5000 Plus from Vistec Lithography. The (100) Si substrate (resistivity = 10^−3^ Ω·cm) is cleaned with UV/ozone and native oxide etched before resist deposition. The e-beam lithography has been optimized by using a 45 nm-thick diluted (3:5 with anisole) PMMA (950 K). For the writing, we use an acceleration voltage of 100 keV, which reduces proximity effects around the dots, compared to lower voltages. We played with different beam currents to expose the nanodots (from 1 nA to 100 nA) as discussed in the paper. Then, the conventional resist development/e-beam Au evaporation (8 nm)/lift-off processes are used. Immediately before evaporation, native oxide is removed with dilute HF solution to allow good electrical contact with the substrate. Single crystal Au nanodots can be obtained after thermal annealing at 260 °C during 2 h under N_2_ atmosphere. At the end of the process, these nanodots are covered with a thin layer of SiO_2_ that is removed by HF at 1% for 1 mn prior to SAM deposition. Spacing between Au nanodots is flexible and is typically set between 50 nm to 200 nm.

### Self-assembled monolayer

As described in [1], for the SAM deposition, we exposed the freshly evaporated gold surfaces and nanodots to 1 mM solution of 11-ferrocenyl-1-undecanethiol (from Aldrich) in 80% ethanol (VLSI grade from Carlo Erba) 20% dichloromethane during 24 h in a glovebox in the darkness. Then, we rinsed the treated substrates with ethanol followed by a cleaning in an ultrasonic bath of chloroform (99% from Carlo Erba) during 1 min.

### XPS

As described in [25], X-ray photoemmission spectroscopy (XPS) measurements have been performed using a Physical Electronics 5600 spectrometer. A monochromatic Al Kα X-ray source (*h*ν = 1486.6 eV) and an analyzer pass energy of 12 eV. The acceptance angle of the analyzer has been set to 14°, the detection angle was 45°, and the analyzed area was deﬁned by an entrance slit of 400 μm.

## Supporting Information

File 1 Detailed code for the “sequence method”.

## References

[1] Clément N, Patriarche G, Smaali K, Vaurette F, Nishiguchi K, Troadec D, Fujiwara A, Vuillaume D (2011). Small.

[2] Lee S H, Jo G, Park W, Lee S, Kim Y-S, Cho B K, Lee T, Kim W B (2010). ACS Nano.

[3] Shin D O, Lee D H, Moon S-J, Jeong S-J, Kim J Y, Mun J H, Cho H, Park S, Kim S O (2011). Adv Funct Mater.

[R4] 4Smaali, K.; Desbief, S.; Foti, G.; Frederiksen, T.; Sanchez, D.; Andres, A.; Leclère, P.; Vuillaume, D.; Clément, N. “On the Mechanical and Electronic Properties of Thiolated Gold Nanocrystals”. To be submitted for publication.

[5] Schaal P A, Simon U (2013). Beilstein J Nanotechnol.

[6] Balcells L, Abad L, Rojas H, Perez del Pino A, Estrade S, Arbiol J, Peiro F, Martínez B (2008). Small.

[7] Mäder T, Höche T, Gerlach J W, Perlt S, Dorfmüller J, Saliba M, Vogelgesang R, Kern K, Rauschenbach B (2010). Nano Lett.

[8] Smaali K, Clément N, Patriarche G, Vuillaume D (2012). ACS Nano.

[9] Jett J E, Lederman D, Wollenberg L A, Li D, Flora D R, Bostick C D, Tracy T S, Gannet P M (2013). J Am Chem Soc.

[10] Wang F, Clément N, Ducatteau D, Troadec D, Tanbakuchi H, Legrand B, Dambrine G, Théron D (2014). Nanotechnology.

[11] Liu P, Sun J, Huang J, Peng R, Tang J, Ding J (2010). Nanoscale.

[12] Palma M, Abramson J J, Gorodetsky A A, Penzo E, Gonzalez R L, Sheetz M P, Nuckolls C, Hone J, Wind S J (2011). J Am Chem Soc.

[13] Pi F, Dillard P, Limouzin L, Charrier A, Sengupta K (2013). Nano Lett.

[14] Guilles S, Winter S, Michael K E, Meffert S H, Li P, Greben K, Simon U, Offenhäusser A, Mayer D (2012). Small.

[15] Huang J-S, Callegari V, Geisler P, Brüning C, Kern K, Prangsma J C, Wu X, Feichtner T, Ziegler J, Weinmann P (2010). Nat Commun.

[16] Barcelo S J, Lam S-T, Gibson G A, Sheng X, Henze D (2012). Proc SPIE.

[17] Park M, Harrison C, Chaikin P M, Register R A, Adamson D H (1997). Science.

[18] Pearson A C, Pound E, Wooley A T, Linford M R, Harb J N, Davis R C (2011). Nano Lett.

[19] Gadegaard N, Thoms S, Macintyre D S, Mcghee K, Gallagher J, Casey B, Wilkinson C D W (2003). Microelectron Eng.

[20] Lacatena V, Haras M, Robillard J-F, Monfray S, Skotnicki T, Dubois E (2014). Microelectron Eng.

[21] Tobing L Y M, Tjahjana L, Zhang D H (2013). Nanotechnology.

[22] Woodbridge C M, Pugmire D L, Johnson R C, Boag N M, Langell M A (2000). J Phys Chem B.

[23] Méndez De Leo L P, de la Llave E, Scherlis D, Williams F J (2013). J Chem Phys.

[24] Umaña M, Rolison D R, Nowak R, Daum P, Murray R W (1980). Surf Sci.

[25] Clément N, Guérin D, Pleutin S, Godey S, Vuillaume D (2012). J Phys Chem C.

