# Supplementary material for: High speed e-beam lithography for gold nanoarray fabrication and use in nanotechnology
Source: Beilstein J Nanotechnol. 2014 Oct 30;5:1918–25. doi: 10.3762/bjnano.5.202 (PMC4222405; doi:10.3762/bjnano.5.202)
Supplement: File 1 — Detailed code for the “sequence method”. [file Beilstein_J_Nanotechnol-05-1918-s001.pdf]

Supporting Information

for

High speed e-beam lithography for gold nanoarray fabrication and use in nanotechnology

Jorge Trasobares*[1], François Vaurette*[1], Marc François[1], Hans Romijn[2], Jean-Louis Codron[1], Dominique Vuillaume[1], Didier Théron[1] and Nicolas Clément[1]

Address: [1]Institut d'Electronique Microélectronique et Nanotechnologie (IEMN) CNRS, Avenue Poincaré, 59652, Villeneuve d'Ascq, France and [2]Vistec Lithography BV, De Dintel 27a, 5684 PS Best, The Netherlands

Email: Jorge Trasobares - jorge.trasobares@ed.univ-lille1.fr; François Vaurette - francois.vaurette@iemn.univ-lille1.fr

* Corresponding author

Detailed code for the "sequence method"

The sequence is defined as a chain of commands to move the beam and expose pixels. The command "jump" is used to move the beam with a step equals to a multiple of the subfield resolution (e.g., 0.5 nm). The exposure of pixels is done by exposing a line of one pixel with the command "Line".

The sequence covers a subfield (e.g., a square of 4 microns) and is repeated to cover the complete main field.

Here is our commented file for the sequence in order to write a triangular array of dots:

	! Start of double column 1
Sequence LineClear X 0 RELJMP	!Start with LineClear: Clear Memory and write a line of (n+1) pixels (n=0 for a single pixel).
Sequence Jump 0 **a**	!jump to the next pixel in Y (**a**: steps of subfield resolution)
Sequence Line X 0 RELJMP	!write a pixel
....	!repeated to fill the subfield in Y to expose the first column
Sequence Jump 0 a	
Sequence Line X 0 RELJMP	
!Top of column 1	
Sequence Jump b c	! jump to top of column 2 X and Y in case of a triangular array (**b and c**: steps of subfield resolution)
Sequence Line X 0 RELJMP	
Sequence Jump 0 -a	
....	! repeated to fill the subfield in Y to expose the first column
Sequence Line X 0 RELJMP	
Sequence Jump 0 -a	
Sequence Line X 0 RELJMP	
!Bottom of column 2	
Sequence Jump b –c	!jump to bottom of column 3
!Start of double column 3	
Sequence Line X 0 RELJMP	
....	!columns repeated to fill the subfield in X
Sequence Jump 0 -a	
Sequence Line X 0	!last pixel to expose in the subfield
	!Bottom of last column
	!complete
	!Then the sequence is repeated to cover a field (i.e. a square of 500 microns)
